# Gamification of Hospital Utilization: Incorporating Cost-consciousness in Daily Practices

**DOI:** 10.7759/cureus.3094

**Published:** 2018-08-03

**Authors:** Peter J Tomaselli, Dimitrios Papanagnou, Jonathan E Karademos, Elizabeth Teixeira, Xiao Chi Zhang

**Affiliations:** 1 Department of Emergency Medicine, Thomas Jefferson University, Philadelphia, USA; 2 Department of Emergency Medicine, Thomas Jefferson University Hospitals, Philadelphia, USA

**Keywords:** healthcare cost reduction, medical education, gamification

## Abstract

Healthcare costs in the United States have skyrocketed over the past decade, contributing to an estimated $750 billion in wasteful spending annually. Despite the demand to improve residency education on value-based, cost-conscious healthcare, there is no consensus on how to best teach this practice. Traditional lectures have failed to demonstrate enduring change in clinical practice patterns, provider attitudes, and reductions in hospital expenditures. We sought to evaluate whether gamification is an effective pedagogical tool to teach cost-consciousness to emergency medicine (EM) residents by creating a 60-minute interactive session based on the popular gameshow, the Price is Right. Costs and associated charges for common laboratory tests, radiographic studies, medications, and common physical resources typically found in the emergency department (ED) were first obtained through direct communication with the ED clinical director and hospital leadership. The session itself consisted of three phases with several Price-is-Right-themed games, which included realistic visual stimuli reminiscent of the gameshow that were created by the authors using the PowerPoint. Formal quantitative and qualitative feedback was solicited at the end of the session. Quantitative evaluation of the educational intervention was obtained through a 22-item questionnaire using a five-point Likert-type scale from 19 of the 22 enrolled residents (86% response rate). Responses were generally very positive with an overall course rating score of 4.16 (SD +/- 0.90). Qualitative feedback identified learners’ predilection for gamified delivery of nonclinical content during conference. The majority of residents (89%) recommend the activity to be used in subsequent offerings to other learners. With healthcare costs on the rise, our feasibility study demonstrated that gamification is an effective way to teach mindful, cost-conscious care to EM residents. Gamification offers a fun and engaging alternative that should be further utilized in EM educational formats. Future studies are needed to longitudinally assess the learner retention and cost-containment practices.

## Introduction

Healthcare costs in the United States have skyrocketed, making the United States one of the few countries with the highest healthcare expenditures in the world. Nearly 18% of the 2016 US gross domestic product (GDP), $3.3 trillion, was spent on healthcare, compared to only five percent, or $27.2 billion spent in 1960 [1]. Several attempts to control the rising costs have included insurance reform, malpractice reform, and prescription reform [2-4]. Other efforts have centered around the development of provider cost-conscious decision rules (i.e., Canadian Head Computed Tomography Rules; Ottawa Ankle Rules). Despite these efforts, however, a number of studies [5-7] have shown that most physicians, particularly resident physicians, are still unaware of the costs of the tests and interventions they routinely order.

As healthcare costs are expected to rise, physicians will require training that will provide them with value-based, cost-conscious practices that can be applied throughout their career (i.e., training them on the financial impact of their clinical decision making) [8]. Emergency medicine (EM) represents an area of particular need for cost education. As of 2012, 20% of payment for emergency department (ED) services was out-of-pocket [9], with the potential of adding a significant financial burden on patients and their families.

Traditional lectures have failed to demonstrate sustainable changes in clinical practice patterns, provider attitudes, and reductions in hospital expenditures [10]. As gamification has shown promise for increasing both medical knowledge and learner engagement [11], the authors propose an interactive learning solution utilizing the popular “The Price is Right” television show to teach cost-conscious care that has been successfully integrated into the EM residency curriculum at the Thomas Jefferson University in Philadelphia, PA. 

## Technical report

Study setting

Learners Targeted: EM Residents

Group size: six to eight learners per group.

Personnel: An EM faculty member presented the activity and, along with three faculty facilitators, provided supervision for the cost game. Faculty members helped keep score; answer questions that came up during the activity; and assisted in determining the items, as well as the actual charges and costs, from the resources obtained from the hospital.

Materials: Whiteboard, dry-erase board (one per group), Powerpoint.

Accreditation Council for Graduate Medical Education (ACGME) Milestones: This activity provides learners with the opportunity to practice systems-based management (i.e., SBP-2, Improving Health Care Delivery and Flow) and patient care (PC-3, Diagnostic Testing). During the activity, learners must demonstrate an awareness of medical costs and an ability to synthesize this knowledge in cost-effective practices (Table 1).

**Table 1:** Emergency medicine-specific Accreditation Council for Graduate Medical Education (ACGME) milestones and learning theories associated with medical cost game activity.

ACGME milestone	Medical cost game activity	Relevant educational learning theories
*PC3*: Diagnostic testing	“Come on Down," “More or Less,” “Cliffhanger”	*Behaviorism*: Positive behavior is reinforced by progression in the medical cost game activities
*SBP2*: Improving healthcare delivery and flow	“Cost-Efficient Dispositions”	*Constructivism*: Players use previous knowledge to construct alternative pathways in patient-disposition

Detailed activity description

**Figure 1:**
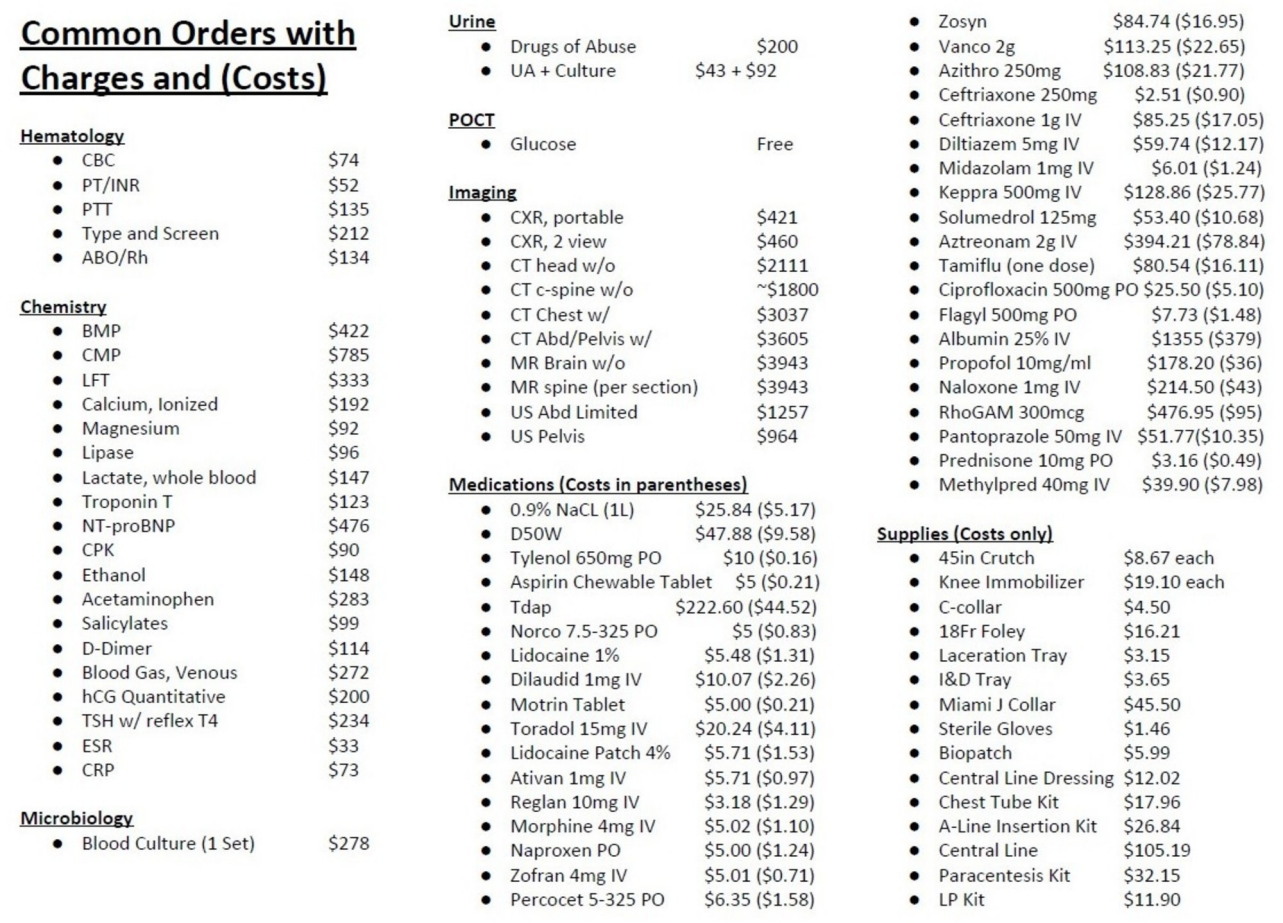
Medical cost and charge examples.

A 60-minute interactive session based on the popular gameshow, The Price is Right, was developed that incorporated concepts from both constructivist and behaviorist theories: residents were able to use their experiences from previous patient management in the emergency department (ED) to navigate decisions and plans that demanded cost-consciousness in hopes of refining their understanding of cost utilization (constructivism) and incrementally build on their existing schemas of healthcare costs to improve performance and foster independent thinking with regards to cost-conscious patient care (behaviorism). The session was incorporated into the EM didactic residency curriculum as one-time special conference didactic. Costs and associated charges for common laboratory tests, radiographic studies, medications, and common physical resources typically found in the ED were first obtained through direct communication with the ED clinical director and leadership from the Departments of Radiology, Pharmacy, Pathology and Supply Chain, respectively (Figure 1). The session itself consisted of three phases with several themed games from the show; realistic visual stimuli reminiscent of the gameshow were created by the authors through the use of the PowerPoint. Learners were not given any medical costs or pre-assignment before the conference didactic.

**Figure 2:**
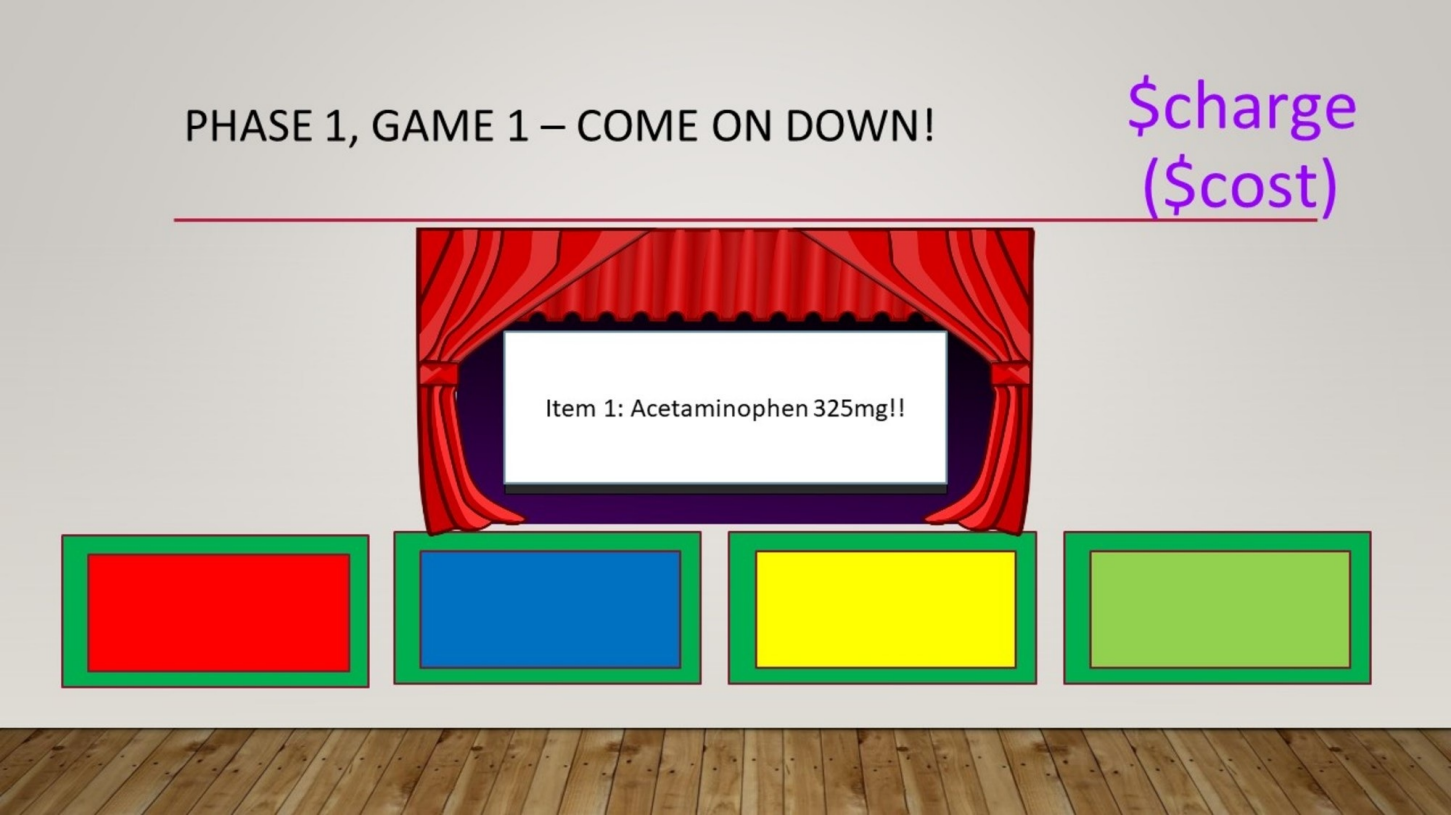
Game 1: Come on Down. How to play: 1) Select one representative from each team. 2) Each representative must guess how much an emergency department study/intervention charges. 3) Order is based on a clockwise manner (everyone gets to guess first and last). 4) The team with the closest guess without going over gets one point.

**Figure 3:**
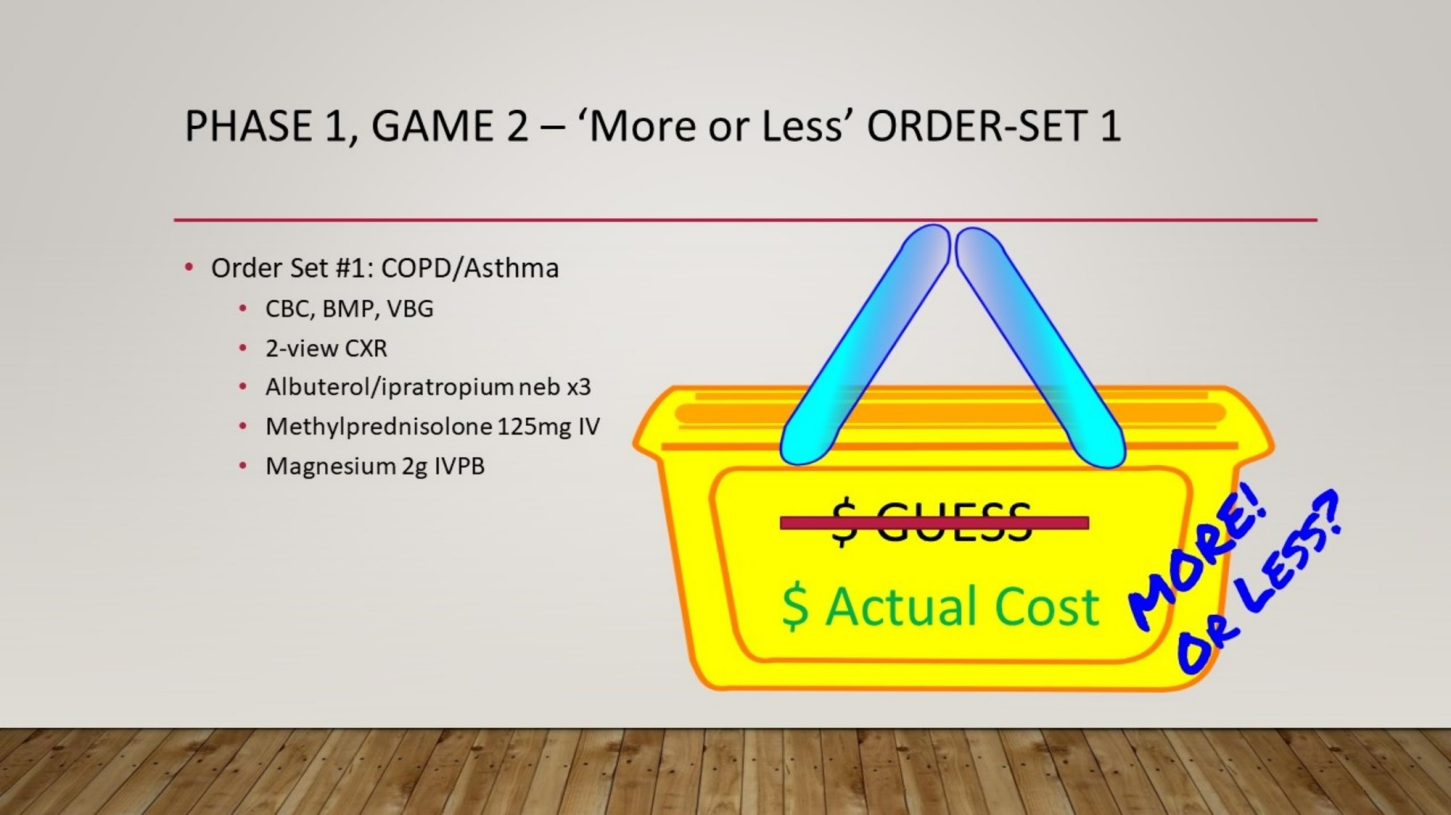
Game 2: More or Less. How to play: 1) Select one representative from each team. 2) Each team is given the cost of an emergency department order set at your institution. 3) Each team must guess whether the stated cost is higher or lower than the cost. 4) The team that guesses correctly gets one point.

**Figure 4:**
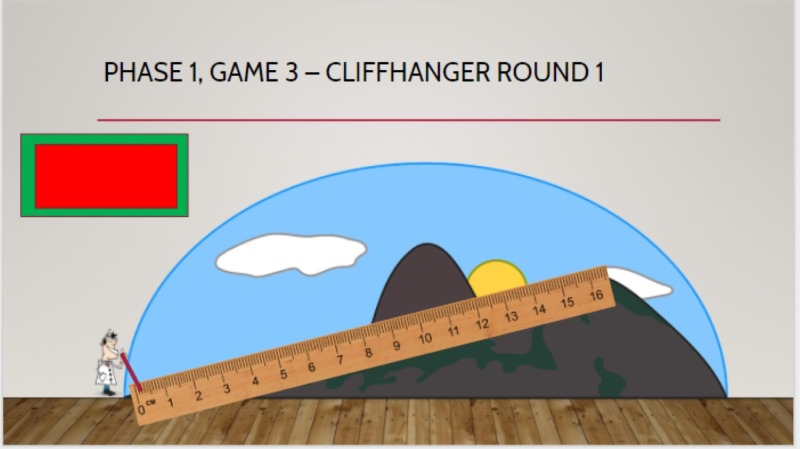
Game 3: Cliffhanger. How to play: 1) Select one representative from each team. 2) Each team must guess the cost of an emergency department item. 3) For each $1 off the actual price, the cartoon doctor will climb one unit. 4) Game ends when the cartoon doctor falls off the cliff. 5) Each item that the team that guesses correctly gets one point.

Phase 1 evaluated the learners’ knowledge of common ED costs with three separate, successive games that were followed by a debriefing that discussed actual dollar values with residents. Game 1 - “Come on Down!” tasked teams to guess the charge for several ED medications and items (Figure 2). Answers were written on small dry-erase boards; the team that made a monetary estimate that was closest to the actual price, without going over, received a point. The actual cost of each medication was shown prior to moving on to the next item. Game 2 - “More or Less” tasked each team to approximate the charge of specific order sets at our institution (i.e., Asthma panels, Chest Pain panels, Gastrointestinal Bleed panels, and Sepsis panels) (Figure 3). An artificial charge was shown for a specific order set, and teams were asked whether the actual charge was “more or less” than this price point. One point was awarded for correct answers. Game 3 - “Cliffhanger” tasked teams to estimate the cost of several common ED supplies and medications priced between $1.00 and $10.00. Each dollar estimate from the actual cost resulted in an animated pixelated PowerPoint character that climbed up a steep ‘cost-cliff’ (Figure 4). After 16 possible “steps,” the character would fall off the cliff, indicating the end of the team’s turn. One point was given to each item that the team was able to correctly estimate the cost for prior to the character falling off the cliff. Each team was given a turn to play this game.

**Figure 5:**
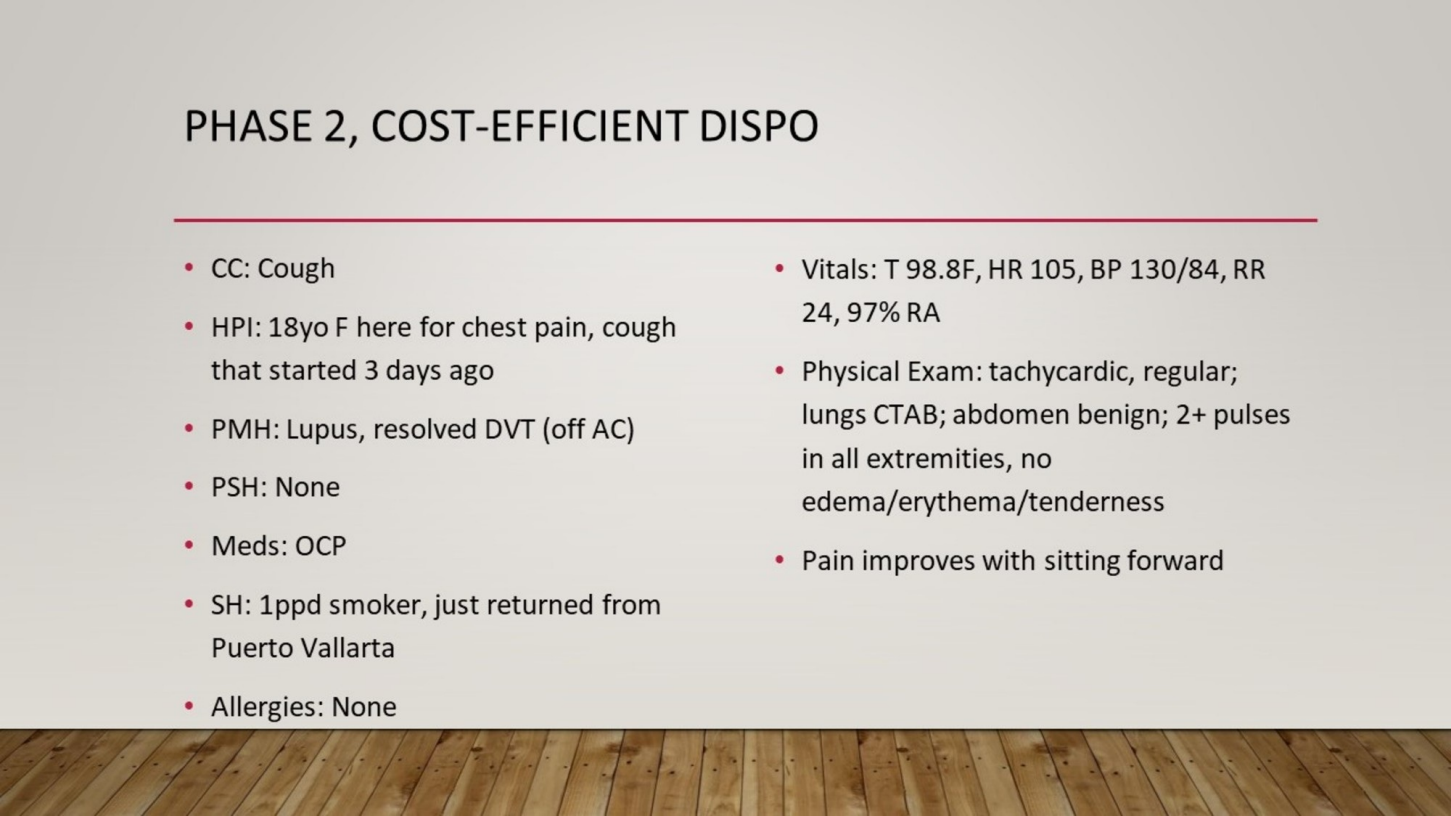
Game 4: Cost-efficient dispo. How to play: 1) All teams will be given a patient encounter. 2) Each team must reach the appropriate disposition by ordering from the available diagnostic or therapeutic emergency department interventions. 3) Upon completion, the team that reaches the correct disposition while spending the least amount ($) will be rewarded by the maximum points (four points). 4) The other teams will be awarded in the reverse order based on their healthcare spending. NOTE: Inappropriate disposition without sufficient workup will not be awarded with any points. Each case will be reviewed by the instructor to see whether there is sufficient workup to warrant a disposition.

Phase 2 challenged learners to practice cost-conscious care on an undifferenced patient while adhering to evidence-based clinical practices (Figure 5). Teams were given a guide sheet that included the charge of the most common orders within the ED. A patient scenario was provided to teams, and members were asked to collectively determine the most cost-efficient disposition, while correctly obtaining the patient diagnosis and adhering to best practices in ED care. During this phase, each team was supervised by an EM faculty member who provided the team with prompts (i.e., lab results, electrocardiograms, imaging) when asked. Points were awarded based on the most cost-effective plan and accuracy of clinical diagnosis.

**Figure 6:**
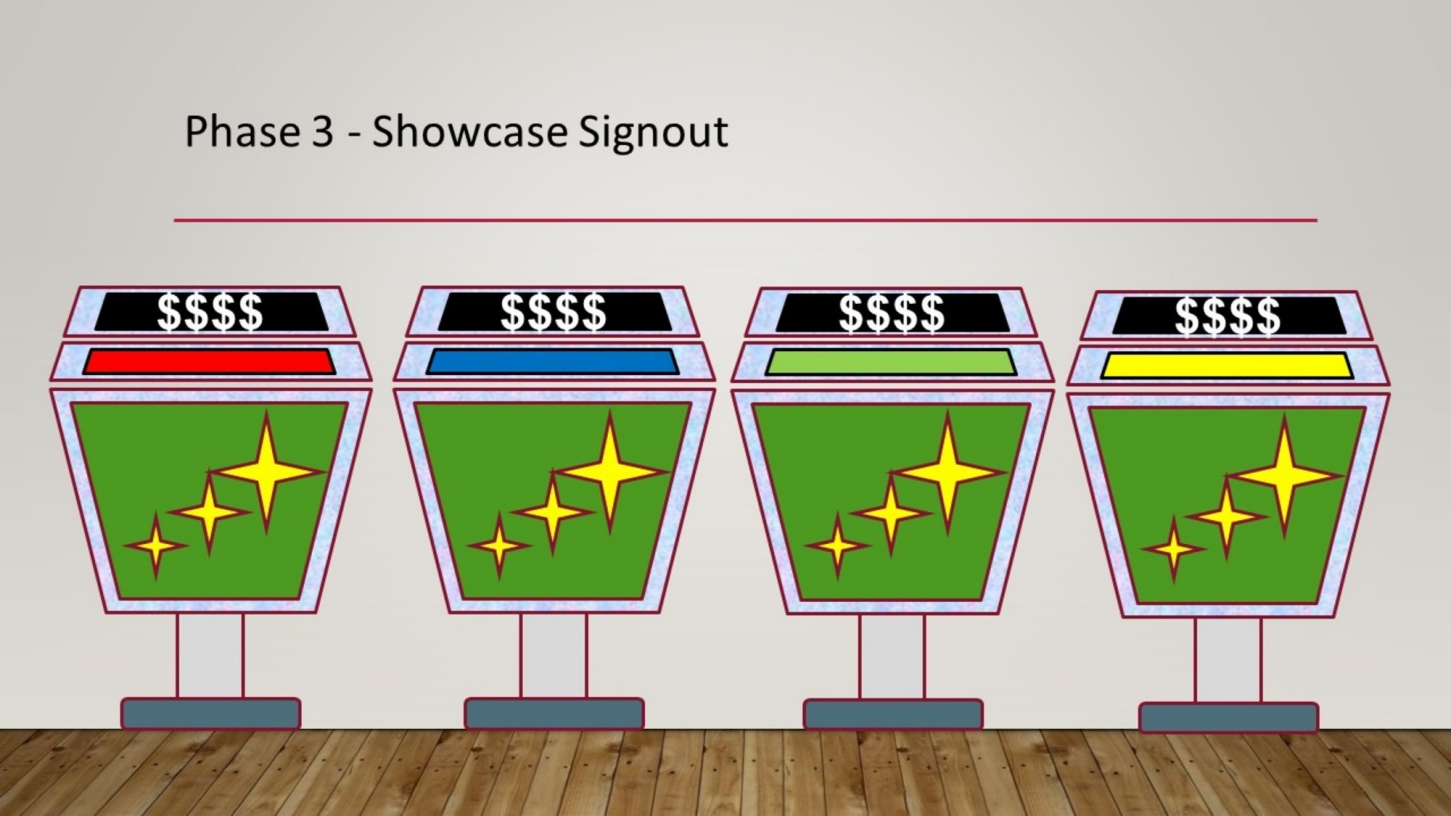
Game 5: Showcase Signout. How to play: 1) Each team must guess the cost of a comprehensive workup (to be revealed) in 60 seconds. 2) The team that guesses the closest to the actual cost without going over will receive five points!!! Bonus: Extra five points for guessing within $1000 of the cost!

Phase 3 concluded with a ‘Showcase Sign-Out,’ where learners were asked to estimate the cost of a comprehensive medical work-up (Figure 6). A final case was given, and detailed a full comprehensive work-up, inclusive of laboratory tests, medications administered, and imaging performed. Adhering to “The Price is Right” rules, the team that made a monetary estimate closest to the actual price of the showcase, without going over, was awarded five points. As an added bonus, any team(s) correctly estimating within $1000 of the amount was/were given an additional five points. Formal quantitative and qualitative feedback was solicited at the end of the session.

Results

**Figure 7:**
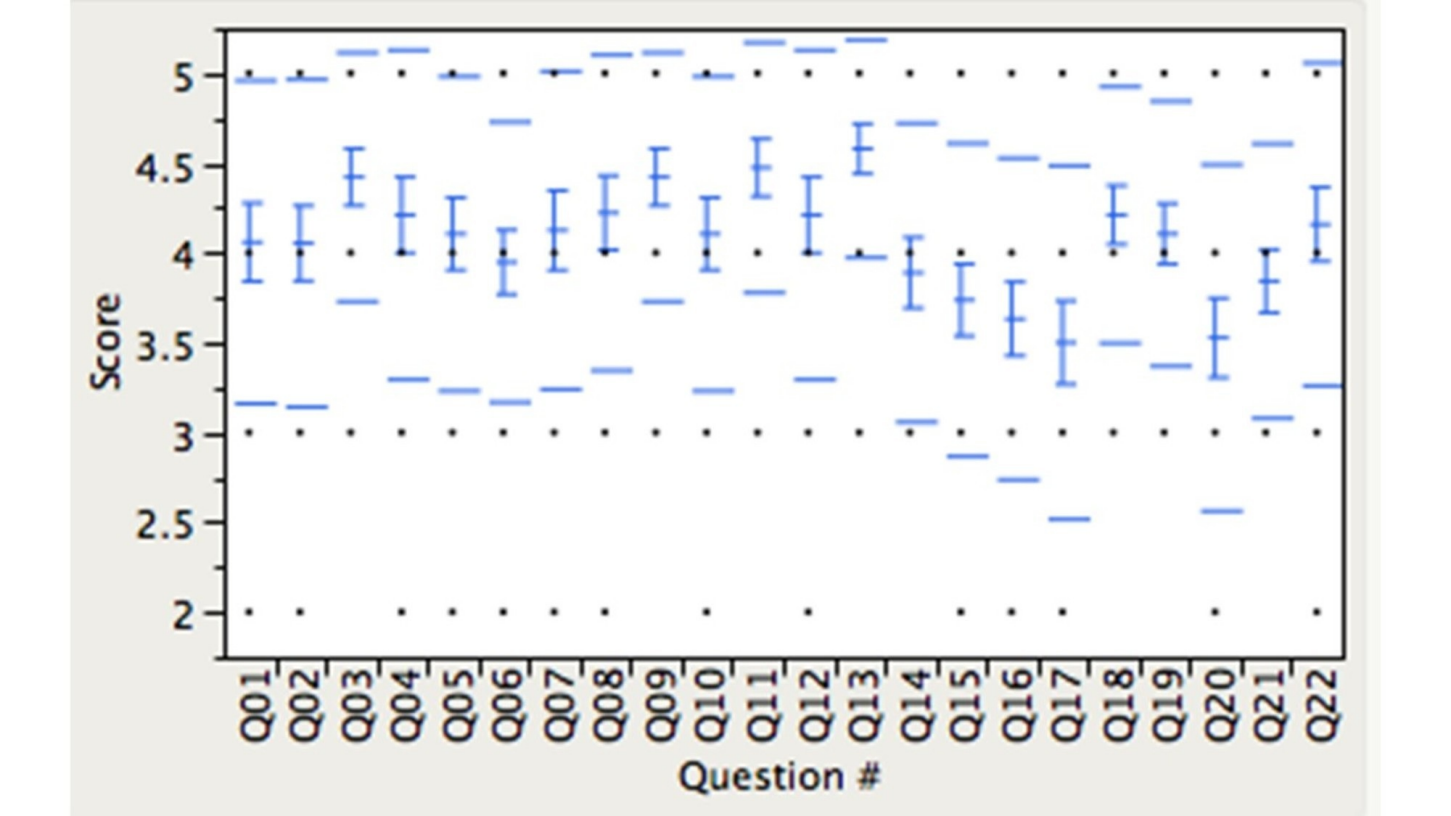
Post-medical cost game survey response. All questions were based on a five-point likert scale (1 = strongly disagree, 5 = strongly agree) or (1 = extremely poor, 5 = excellent). Detailed questions are listed in Appendix 1.

Quantitative evaluation of the educational intervention was obtained through a 22-item questionnaire using a five-point Likert scale from 19 of the 22 enrolled residents (86% response rate). Responses were generally positive, with an overall course rating score of 4.16 (SD +/- 0.90) (Figure 7). Nearly all learners reported increased knowledge of hospital costs (4.58 +/- 0.61); understanding of cost concepts (4.42 +/- 0.69); and insight into increased spending (4.21 +/- 0.71). Learners reported that the session was structured in a sequence that was logical and easy to follow (4.05 +/- 0.90), and that the interactive nature of the game complemented their existing knowledge on cost practices (4.47 +/- 0.68). Qualitative feedback identified learners’ predilection for gamified delivery of nonclinical content during the conference. The majority of residents (89%) recommend the activity be used in subsequent offerings for other learners. During the phase 1 debriefing, learners consistently expressed surprise at the high costs of testing and medications. This led to more questions from learners regarding additional items not included in phase 1. Ultimately it provided a great transition to phase 2 where the additional costs of items were revealed.

## Discussion

The design of our activity allowed us to first educate residents about the costs of individual tests, medications, and healthcare supplies. The repetition of prices, the surprise many residents felt regarding the cost of commonly ordered tests, and the points awarded for correct responses employed a behaviorist model for the most basic level of understanding. As we progressed to Phase 2, a constructivist approach was utilized, with residents synthesizing both the knowledge gained in Phase 1 with their prior medical knowledge and direct clinical experience to formulate responsible, cost-efficient plans. These learning objectives are in line with the ACGME objectives for diagnostic studies (PC3) and systems-based management (SBP2), with strong scores in increased knowledge of hospital cost and clearer understanding of cost concepts.

Over the course of this activity, the authors noted that the residents who were highly interactive during the game appreciated the educational session the most. This was contingent on learners suspending their preconceived notions about healthcare costs and fully immersing themselves in the activity. 

The learner to faculty ratio was approximately seven to eight students per faculty. We felt this ratio was crucial for the overall success of this didactic as it allowed multiple residents from each training level to provide their own perspectives and receive faculty input within the allotted time. Allocating the correct amount of time can be challenging with these types of lectures. Insufficient or overabundant time allotted per lecture can result in the failure to cover all necessary materials or failure to capture the learners' attention, respectively. While 60 minutes were allocated for this lecture, we believe that future iterations should allow for at least 70 minutes to allow for smoother transitions between each phase as well as time to field additional questions. The medical cost handout provided to learners during Phase 2 was very well received as it decreased the cognitive load when calculating the total cost of the workup and served as a convenient take-home handout due to its convenient formatting that reflected the existing order panels for the most commonly ordered ED items.

The study was limited in power, as it was conducted at a single center with a small sample size of EM learners, with cost and charges only applicable to the authors' home institution. Furthermore, the authors were only able to provide paired cost and charges of limited items in the ED. While the authors would have liked to demonstrate how hospital charges varied with insurance companies and individualized plans, the overarching goal was to determine if utilizing a game to reflect on healthcare costs was feasible in EM resident learners. The authors posit that the proposed structure described in this educational technical report can be easily generalized to other institutions with their respective costs and charges.

## Conclusions

With healthcare costs on the rise, our feasibility study demonstrated that gamification is an effective way to teach mindful, cost-conscious care to EM residents. Gamification offers a fun and engaging alternative that should be further utilized in EM educational formats. Future studies are needed to longitudinally assess learner retention and cost-containment practices.

## Appendices

Appendix 1: Post Medical Cost Game Evaluation Survey. Questions 1-22 were evaluated based on a 5-point Likert scale (1=strongly disagree, 5=strongly agree) or (1=extremely poor, 5=excellent).

1.    The course presented skills in a helpful sequence

2.    The course provided an appropriate balance between instruction and practice

3.    The course was appropriate for the stated level of the class

4.    The course was organized in a way that helped me learn

5.    The course provided a mixture of explanation and practice

6.    The course was effectively organized

7.    The course lectures complemented the EM curriculum

8.    The course instructions (including Powerpoint directions and verbal instructions) were clear

9.    The course helped me understand concepts (i.e. cost-conscious care) more clearly

10.   Instructions for the game content were clear

11.   The interactive game complemented my understanding of the lectures

12.   The section helped to complement the residency training

13.   Increased knowledge in hospital cost

14.    The course provided guidance on how to become a competent professional

15.    The course developed my ability to order hospital tests critically

16.   The course  helped me improve the way I order tests for workup

17.    The course developed my ability to provide constructive critiques to others

18.    The course gave me a deeper insight into the topic

19.    In this course, I learned a great deal

20.    The course improved my problem-solving skills

21.    The course developed my ability to think critically about the subject

22.    Overall, I would rate this presentation/instruction as:
